# Weed25: A deep learning dataset for weed identification

**DOI:** 10.3389/fpls.2022.1053329

**Published:** 2022-11-30

**Authors:** Pei Wang, Yin Tang, Fan Luo, Lihong Wang, Chengsong Li, Qi Niu, Hui Li

**Affiliations:** ^1^ Key Laboratory of Agricultural Equipment for Hilly and Mountain Areas, College of Engineering and Technology, Southwest University, Chongqing, China; ^2^ Key Laboratory of Modern Agricultural Equipment and Technology (Jiangsu University), Ministry of Education, School of Agricultural Engineering, Jiangsu University, Zhenjiang, China; ^3^ Interdisciplinary Research Center for Agriculture Green Development in Yangtze River Basin, Southwest University, Chongqing, China; ^4^ National Citrus Engineering Research Center, Chinese Academy of Agricultural Sciences and Southwest University, Chongqing, China

**Keywords:** Weed25, weed dataset, deep learning, weed identification, weed species

## Abstract

Weed suppression is an important factor affecting crop yields. Precise identification of weed species will contribute to automatic weeding by applying proper herbicides, hoeing position determination, and hoeing depth to specific plants as well as reducing crop injury. However, the lack of datasets of weeds in the field has limited the application of deep learning techniques in weed management. In this paper, it presented a dataset of weeds in fields, Weed25, which contained 14,035 images of 25 different weed species. Both monocot and dicot weed image resources were included in this dataset. Meanwhile, weed images at different growth stages were also recorded. Several common deep learning detection models—YOLOv3, YOLOv5, and Faster R-CNN—were applied for weed identification model training using this dataset. The results showed that the average accuracy of detection under the same training parameters were 91.8%, 92.4%, and 92.15% respectively. It presented that Weed25 could be a potential effective training resource for further development of in-field real-time weed identification models. The dataset is available at https://pan.baidu.com/s/1rnUoDm7IxxmX1n1LmtXNXw; the password is rn5h.

## Introduction

Weed suppression is one of the greatest factors affecting crop production. The weeds could compete with crops for water, light, fertilizer, growth space, other nutrients, *etc*., resulting in reduction of crop yield and production quality (Khan et al., 2021). It could also be the host of many pathogens and insects, which would damage crop plants. According to a survey, the worldwide annual loss of crop production caused by weed suppression was 13.2%, which was equivalent to the annual food ration for one billion human beings (Yuan et al., 2020). Thus, weed control plays a vital role in crop management and food security.

Common weed control methods include manual, biological, chemical, and mechanical weeding, *etc*. (Marx et al., 2012; Stepanovic et al., 2016; Kunz et al., 2018; Morin, 2020; Andert, 2021). Manual weeding provides the most precise management of weeds in the field. However, the labor intensity and cost are too high to make it feasible for large-scale cultivation. Biological weeding is safe and friendly to the environment as it brings little injury to non-target organisms, while it usually requires a long period to rebuild the eco-system. Chemical weeding is the most common approach of weed control, mainly through spraying of chemical herbicides. However, the overuse of herbicides has caused many issues, such as environmental pollution, pesticide residues, and weed resistance. According to the survey, 513 biotypes of 267 species of weeds have developed resistance to 21 types of herbicides in various cropland systems (Heap, 2022). Thus, the application of technologies such as precise spraying or mechanical weed management on specific weeds will be of great significance to avoid the over-input of herbicide. Due to the concept of organic agriculture, automatic mechanical weeding is gradually attracting more attention (Cordill and Grift, 2011). It realized weed control without chemical input and saved much fuel as unnecessary tillage could be avoided. However, as the weed identification accuracy is not high enough, unexpected damage to the plant–soil system has been one of the most important barriers to the application of intelligent mechanical weeding (Swain et al., 2011; Gašparović et al., 2020). Therefore, it is imperative to improve the identification accuracy of weeds in the fields.

In weed identification research, several traditional machine learning methods have been applied based on image processing techniques, including support vector machine (SVM) (Bakhshipour and Jafari, 2018), decision tree (Bakhshipour and Zareiforoush, 2020)-based random forest algorithm (Gašparović et al., 2020), and K-nearest neighbor (KNN) classifiers (Pallottino et al., 2018). In these algorithms, the color, texture, shape spectrum, and other characteristics of weed images should be extracted with complex hand-crafting. Thus, similar weed species could not be distinguished if the weed image extraction was incomplete or if there were occluded features.

In 2006, Hinton et al. (2006) proposed the concept of deep learning, pointing out that the structure of deep learning networks was deep and closely connected. The larger datasets would be trained by increasing the speed of algorithms. In recent years, deep learning technology is developing rapidly, showing high accuracy and robustness in the field of image identification (Peteinatos et al., 2020). In particular, ImageNet, a large-scale, multi-variety dataset containing 3.2 million images, presented that large-scale datasets played an important role in improving the identification accuracy of the trained models using deep learning algorithms (Russakovsky et al., 2015). However, both the image amount and the weed species of existing datasets for deep learning-based weed identification model training are in a small scale.

In practice, the weeds should be controlled at the growth stage between three and six leaves so that the crops could occupy the dominance in further growth competition. Conventional algorithms for weed identification used image processing technology to extract the image features of weeds, crops, and background. Bakhshipour et al. (2017) presented a model to distinguish sugar beets and weeds by using wavelet texture features. The principal component analysis was used to select 14 of the 52 extracted texture features. It demonstrated that wavelet texture features could be effectively distinguished between crops and weeds despite many occlusions and overlapping leaves. The color feature-based models could only identify crops and weeds that had obvious differences of pixel values in RGB matrix or other parameter matrixes generated from that. Generally, the color feature was applied in combination with other features—for example, Kazmi et al. (2015) proposed a method which fused surface color and edge shape for leaf detection and vegetation index integration. The vegetation index was integrated into local features by obtaining the accuracy of 99.07%. However, although conventional image processing methods could distinguish weeds and crops, it was difficult to distinguish the weeds in different species.

Deep learning network can form abstract high-level attributes, which will benefit weed identification, rather than the conventional machine vision network using low-level attributes such as color, shape, or texture. As is known, deep learning technique has improved in terms of accuracy and generalization capabilities in the current target detection models. The prevalent target detection networks are composed of Faster R-CNN, Single Shot Detector, You Only Look Once (YOLO) model, *etc*. (Redmon et al., 2016; Ren et al., 2017; Quan et al., 2022). Dyrmann et al. (2016) used convolutional neural networks to identify 22 different plants with a total of 10,413 images. The result showed that the higher classification accuracy took place in the weed species which consisted of a larger number of image resources. Thus, weed identification based on deep learning technology requires sufficient datasets.

There have been some large datasets for object detection model training, such as PASCAL VOC (Everingham et al., 2010), ILSVRC (Russakovsky et al., 2015), COCO (Lin et al., 2014), *etc*. Nevertheless, most of the large and open-access datasets consisted of objects in common life—for example, the PASCAL VOC was composed of 24,000 images in 20 categories such as cats, dogs, cars, *etc*. However, relevant weed datasets were not involved. Many scholars have created some weed datasets for the identification of weeds in specific plots, which usually contained just a few categories and were in small scales. Haug and Ostermann (2015) collected and produced a labeled and available dataset with 60 images at the Organic Carrot Farm. Dyrmann et al. (2016) collated images consisting a total of 10,413 images of 22 crops and weeds from six different datasets in the earlier periods, with an average of 400 images per species. Giselsson et al. (2017) published a dataset of about 960 plants from 12 plant species at different growth stages. Jiang et al. (2018) established a dataset of four species of weeds with 1,200 images for each. Peng et al. (2019) extracted 1,000 images of weeds associated with cotton fields from the video for research, including goosegrass, purslane, and nutgrass. Meanwhile, most of these datasets are not open-access. Olsen et al. (2019) gathered a total of 17,509 labeled datasets of eight species of weeds from Australian ranches, DeepWeeds, which was a large and publicly available dataset of pasture weeds. Sudars et al. (2020) presented a public dataset including 1,118 images of six food crops and eight weeds. On average, each category contained 80 images. Tretyakova et al. (2020) sorted out a plant dataset containing 24,284 images of 329 plant species, with an average of 73 images for each category, which mainly incorporated grain, spring and winter crops, economic crops, and weeds, so it was not a purely weed dataset. Khan et al. (2020) proposed a study of four publicly available datasets, including the Rice Sowing and Weed Dataset, the BoniRob Dataset, the Carrot Crop and Weed Dataset, and the Rice Millet Dataset. In order to quickly identify cotton field weeds, Fan et al. (2021) collected 4,694 pictures in a cotton field including seven types of associated weeds, such as field thistle, crabgrass, and purslane. The datasets are valuable and available for testing algorithms. However, most of them only cover specialized crops or weeds, which are often limited to specific growth stages. Meanwhile, in view of the current research on weed detection in farmlands, many researchers tried to cultivate some small samples in the laboratory and expanded the data through data enhancement, mainly by expanding, enlarging, shrinking, and rotating the original image. Thus, there is currently a lack of open-access large weed datasets.

**Figure 1:**
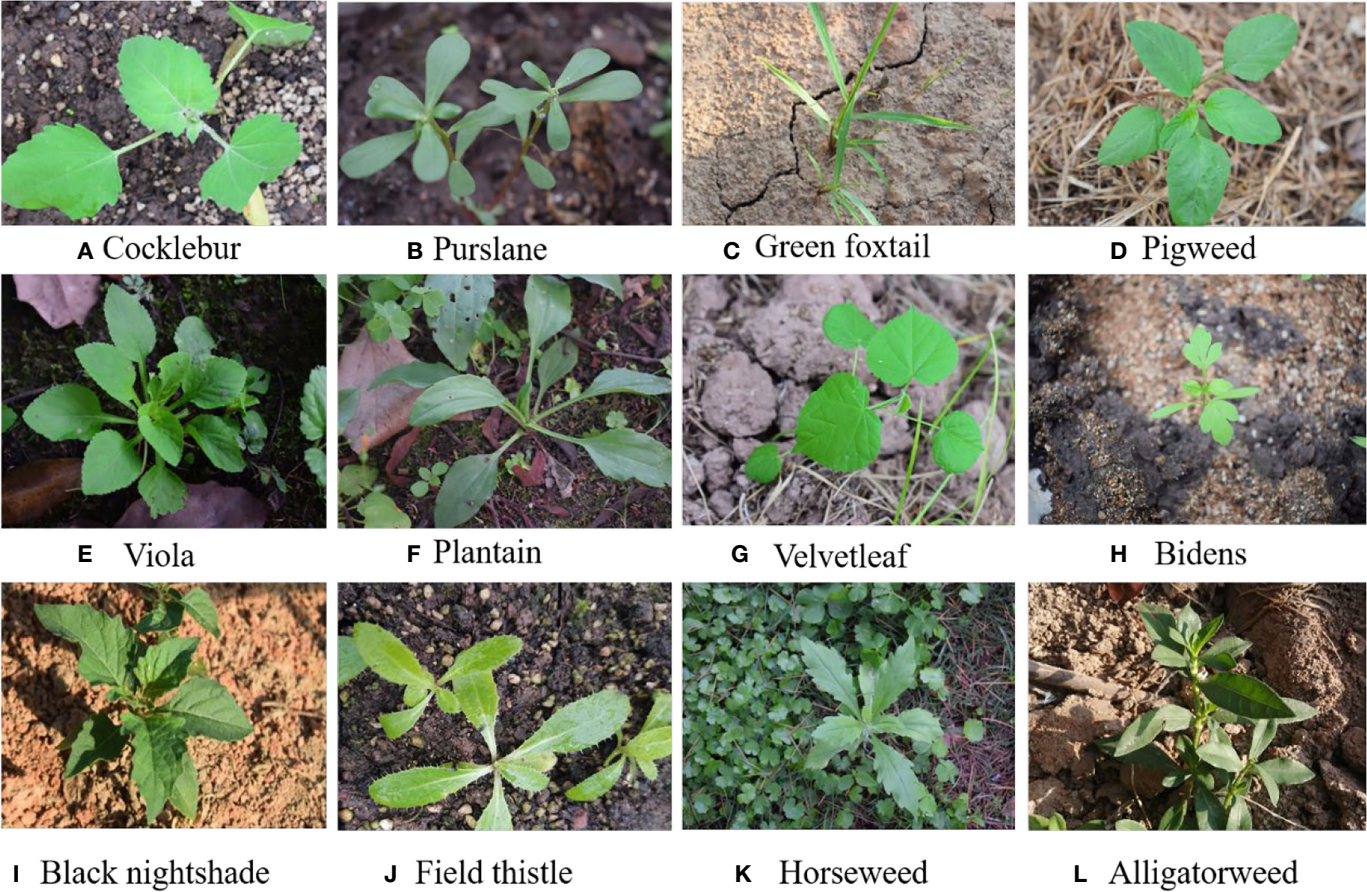
Images of partial samples in the dataset.

To enable better training resources for applying computer vision technology in weed identification, we provided the dataset Weed25 in this paper. This dataset contains 14 families, including 25 species of weeds. The image amount of each weed species was nearly 500–550. It could meet various training requirements for either classification or detection models. Some of the weed images in Weed25 are shown in **Figure 1**. Compared to the farmland weed dataset in the existing literature, the Weed25 dataset is larger and more diverse. Due to the long period of collection, the morphology of a variety of weeds at different growth periods was included. The hypothesis is that, with Weed25, the identification accuracy would be significantly improved using the common deep learning training model.

## Materials and methods

This section mainly introduced the data acquisition, classification, and labeling of the Weed25 dataset.

### Image acquisition

The image resources of Weed25 were acquired from fields and lawns in Chongqing, China, on 25 weed species which are prevalent in East Asia. The images were taken between October 2021 and August 2022. Images were taken at a height and angle of approximately 30–50 cm and 60°–90°, respectively, with a digital camera (Nikon D5300 SLR, Japan) or a smartphone (Huawei Enjoy 9S, China), which means that the shooting angle was as vertical to the weed as possible.

**Figure 2:**
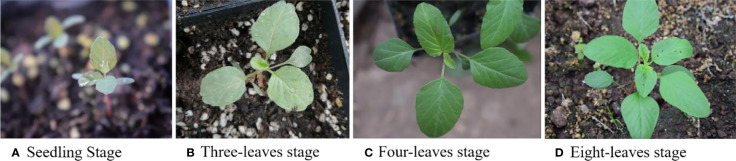
Morphological appearance of purslane at different growth stages.

As sunlight intensity and angle would have impacts on the accuracy of subsequent weed identification, the weed images were taken at different time points—between 9:00 and 17:00—on sunny, cloudy, and rainy days, respectively. Therefore, the light conditions of this weed dataset could represent that in the natural complex environment. In practice, the issues of mutual occlusion and interweaving of weed leaves could bring difficulty to the image acquisition. Meanwhile, to collect the images of weeds at different growth stages, some species of the weeds were selected for greenhouse cultivation. Majority of the weed images were collected when they were at the growth stage of two to nine leaves (BBCH 12–19). Pictures of pigweed at seedling stage, three-leaves stage, four-leaves stage, and eight-leaves stage, respectively, are presented in **Figure 2**. It could be seen that there were significant differences in the morphology of this species of weed at different growth stages.

**Figure 3:**
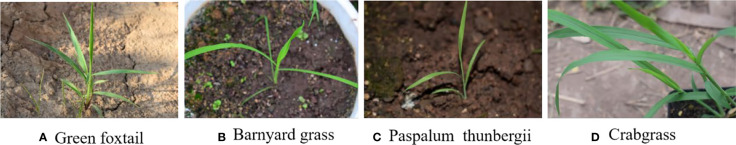
Similarity of grass weeds in the field.

Most species of grass weeds in the field are very similar in appearance and shape, such as barnyard grass, green foxtail, crabgrass, and other *Gramineae* plants, as shown in **Figure 3**. The unique color and similar characteristics of weeds in terms of shape will bring some difficulty in identifying the weeds.

### Classification of dataset structures

**Figure 4:**
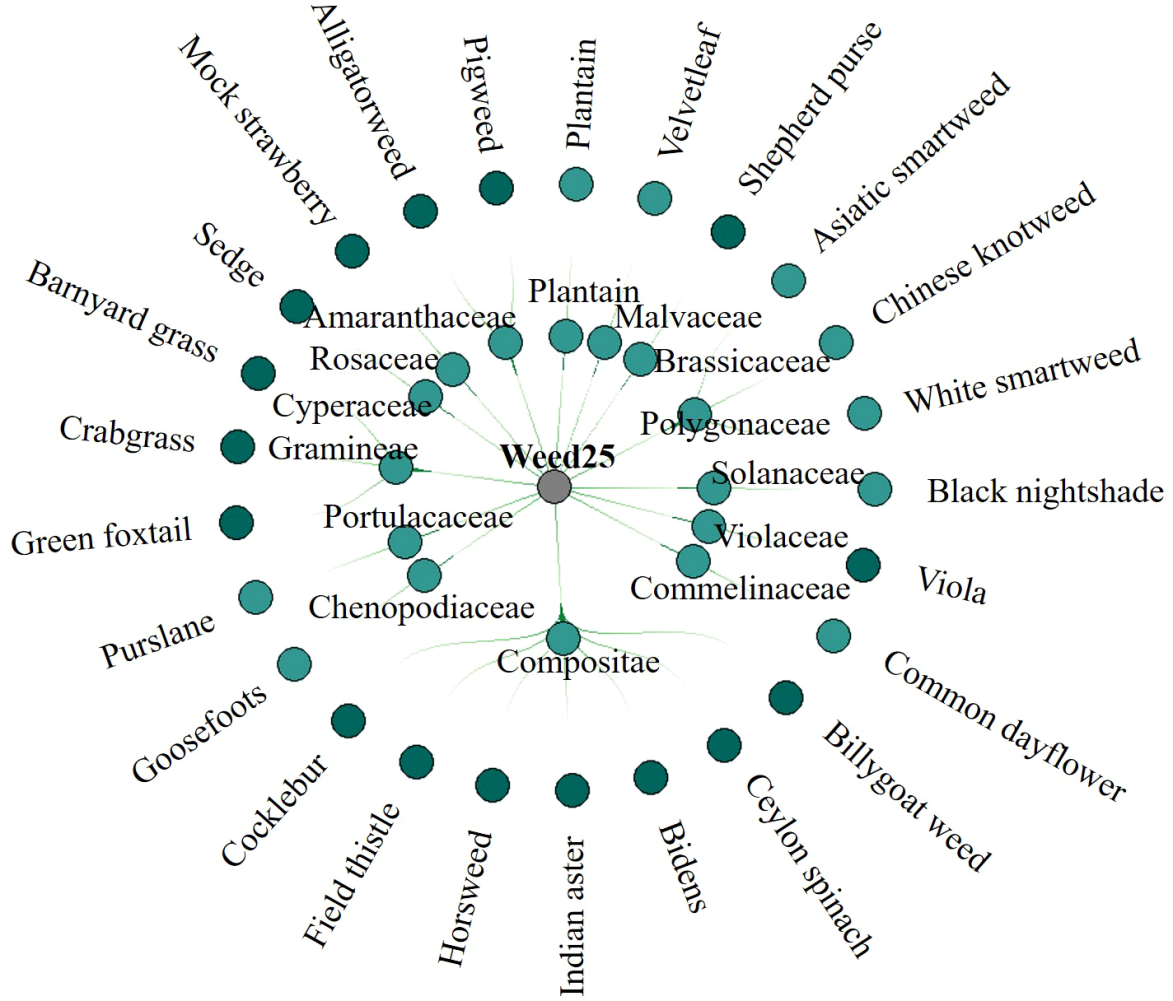
Classification structure hierarchy of Weed25 dataset. The dataset includes 25 weed species from 14 families.

The classification of weeds in Weed25 was conducted mainly with reference to *Primary color ecological map for identification and control of weeds in farmland* (Ren et al., 2018) and *Farmland Weed Identification Primary Color Atlas* (Hun et al., 2012). Weed25 consisted of 25 weed species from 14 families. Because each family was made up of many species of weeds, the different families were classified as a general family. The different weeds under each family were classified as a specific species of the general family—for example, Weed25 was mainly composed of barnyard grass of *Gramineae*, billygoat weed and cocklebur of *Compositae*, and pepper grass of *Cruciferaceae. Gramineae*, *Asteraceae*, and *Cruciferaceae* were the general families in this classification system. The specific weed included in the classification belonged to a general family. The main hierarchy is shown in **Figure 4**. The main occurrence areas and crops of these weeds are summarized and listed in **Table 1**.

**Table 1:** Some of the main crops with weed growth characteristics and hazards.

Family	Species	Features	Main distribution area	Seasons that occur	Main crops
Gramineae	Barnyard grass	Strong vitality and reproductive ability, salt resistance	Temperate regions of the world	Spring, summer, and autumn	Rice, wheat, *etc*.
	Crabgrass	Tenacious vitality and strong reproductive ability	Australia, Argentina, Vietnam, China, *etc*.	Spring, summer, and autumn	Corn, soybeans, cotton, peanuts, tobacco, sugarcane, sorghum, *etc*.
	Green foxtail	The root system is well developed. The ability to absorb soil moisture and nutrients is strong	Temperate, subtropical, and tropical on all continents	Summer and autumn	Millet, corn, sorghum, wheat, soybeans, cotton, vegetables, fruit trees, and other dry crops
Cyperaceae	Sedge	It prefers shade and has strong moisture resistance	China, Australia, Vietnam, Philippines	Spring, summer, and autumn	Rice
Compositae	Cocklebur	The root system is developed. The regeneration ability is strong	Canada, USA, Mexico, Australia, China	Summer and autumn	Corn, cotton, soybeans, peanuts, *etc*.
	Billygoat weed	Strong branching, warm and hardy, easy to spread	Africa, India, Cambodia, Vietnam, and China	Spring, summer, and autumn	Corn, sugar cane, sweet potatoes, *etc*.
Portulacaceae	Purslane	Drought tolerant and flood tolerant	Temperate and tropical around the world	Spring and summer	Vegetable gardens, farmland, *etc*.
Prescarts	Plantain	Hardiness and drought tolerance, can be used medicinally	Japan, Nepal, Malaysia, China, *etc*.	Spring, summer, and autumn	Corn
Chenopodiaceae	Goosefoots	Can be used medicinally	Temperate and tropical around the world	Spring, summer, and autumn	Wheat, cotton, beans, potatoes, peanuts, corn
Commelinaceae	Common dayflower	It prefers temperature and humidity and is highly resistant to drought	China, Vietnam, North America, Russia, *etc*.	Spring, summer, and autumn	Soybeans, wheat, corn, peanuts
Polygonaceae	White smartweed	Strong adaptability; can be used in medicine	China, North Korea, Japan, India, *etc*.	Spring, summer, and autumn	Corn, rice, wheat, beans, potatoes, shallots, cotton, sesame, *etc*.
Amaranthaceae	Pigweed	Adaptable, shade intolerant, edible	All over the world	Spring, summer, and autumn	Cotton, peanuts, beans, potatoes, vegetables, and other dry crops
Brassicaceae	*Rorippa globosa*	The edges of the leaves are jagged and do not require much soil	Temperate regions of the world, China	Spring, summer, and autumn	Corn, beans, potatoes, *etc*.
	Pepper grass	The growing environment is harsh	Asia, Europe, Africa, North America	Spring, summer, and autumn	Winter wheat

The information was mainly derived from Ecological management of agricultural weeds (Liebman et al., 2001), Primary color ecological map for identification and control of weeds in farmland (Ren et al., 2018), and Primary color atlas of farmland weed identification (Hun et al., 2012)].

### Data annotation

All images in Weed25 were manually annotated and verified by three weed scientists. LabelImg was selected as the annotated software, which was a visual graphical image annotation tool created in Python environment. The labels were generated as COCO files for further training.

### Description and division of datasets

The Weed25 dataset contained 14,023 images in 25 categories, which was more diverse than the existing dataset, with most of the weed images collected from the field. The collected weed dataset was divided into training, validation, and test datasets with a ratio of 6:2:2. Specifically, all images of Weed25 were divided into 8,409 training images, 2,807 validation images, and 2,807 testing images, as shown in **Table 2**. For object detection, all dataset images labeled were divided into 9,955 images as the training dataset and 4,068 images as the validation dataset.

**Table 2:** Division of training/validation/testing (denoted as Train/Val/Test) datasets.

Family	Species	Amount	Train	Val/Test
Gramineae	Barnyard grass	563	337	112
	Crabgrass	594	356	118
	Green foxtail	552	331	110
Cyperaceae	Sedge	594	356	118
Compositae	Horseweed	192	115	38
	Field thistle	565	339	339
	Cocklebur	745	447	447
	Indian aster	510	305	306
	Bidens	612	367	367
	Ceylon spinach	536	321	322
	Billygoat weed	599	359	359
Polygonaceae	White smartweed	671	402	134
	Asiatic smartweed	490	294	98
	Chinese knotweed	390	234	78
Amaranthaceae	Alligatorweed	637	381	118
	Pigweed	742	444	127
Brassicaceae	Shepherd purse	224	131	46
Portulacaceae	Purslane	730	438	146
Commelinaceae	Common dayflower	562	337	112
Chenopodiaceae	Goosefoots	593	355	118
Plantain	Plantain	556	333	111
Violaceae	Viola	523	313	104
Solanaceae	Black nightshade	606	363	121
Rosaceae	Mock strawberry	615	369	123
Malvaceae	Velvetleaf	622	373	124
Weed25	–	14,023	8,409	2,807

### Comparison with other datasets

To show the advantages of Weed25 in terms of species diversity and species average (species diversity: the number of all weed species in the dataset, abbreviated as diversity, that was characterized in this paper by the number of species; species average: the mean of the image number of each weed, abbreviated as diversity average, that was used in this paper to characterize the average number of weeds), Weed25 was compared with several existing datasets related to weed identification, as shown in **Table 3**. In terms of diversity, the largest dataset (Tretyakova et al., 2020) was comprised of 329 categories, while the smallest dataset (Jiang et al., 2018) had only four categories. Although the dataset created by Tretyakova et al. (2020) contained 329 categories, the images were not only weeds but also plants such as grains and economic crops. The species average of this dataset was 73, which was usually not sufficient for model training. Moreover, most datasets were not open-access (Jiang et al., 2018; Sudars et al., 2020; Tretyakova et al., 2020). Majority of the existing datasets had a certain imbalance on species diversity and evenness (Giselsson et al., 2017; Sudars et al., 2020; Fan et al., 2021), which led to difficulties in practical applications. According to the previous survey, we created Weed25 in the fields such as farmland and lawn. The diversity and average should be more reasonable compared with the existing weed datasets.

**Table 3:** Weed25 compared with other weed datasets.

Authors	Year	Species	Number	Average	Illustration
Tretyakova et al., 2020	2020	329	24,284	73	Datasets of plants such as grains, crops, and weeds
Jiang et al., 2018	2018	4	4,800	1,200	Cornfield weeds
Sudars et al., 2020	2020	14	1,118	80	Includes six food crops and eight weeds
Fan et al., 2021	2021	7	4,694	670	Cotton field weeds
Giselsson et al., 2017	2017	12	960	80	Laboratory cultivated
Dyrmann et al., 2016	2016	22	10,413	400	Collated six early datasets, including 22 crops and species
Haug and Ostermann, 2015	2015	–	60	–	Carrot farm weed dataset
Olsen et al., 2019	2019	8	17,509	2,188	Ranch weed dataset
Peng et al., 2019	2019	–	1,000	–	Cotton field weeds
Weed25	2022	25	14,035	561	Datasets for different growth stages in farmland, lawns, and laboratories

## Evaluation test

To verify whether the image in Weed25 could be applied for weed identification model training, several deep learning detection networks were employed for the model training with this dataset.

### Training platform

The device for deep learning model training was a desktop workstation with a processor of Ryzen threadripper 3970x 32-core processor ×64 (AMD®, California, USA). The running memory was 32G. The graphics processing unit was GeForce RTX3060ti (NVIDIA Corporation, Santa Clara, CA, USA). The Pytorch deep learning framework that supports numerous neural network algorithms was processed under Ubuntu20.4.

### Evaluation indicators

In this study, the precision (P), recall (R), and mean average precision (mAP) were used as the evaluation indexes of the trained target detection models. The value range of the three indexes is [0, 1]. Meanwhile, the average of the harmonization of precision and recall (F1 score) was also introduced to reconcile the average evaluation, where precision represents the ratio between the number of correctly detected weeds and predicted weeds of a certain species. Recall represented the proportion of targets for a class of weeds in the sample that were correctly predicted. The specific evaluation calculation formula is as follows:


(1) $$P=\frac{TP}{TP+FP}\times 100\%$$

(2) $$R=\frac{TP}{TP+FN}\times 100\%$$

where TP represents the number of samples correctly divided into positive samples, FP represents the number of incorrectly divided positive samples, and FN represents the number of incorrectly divided negative samples.

The average precision indicates the detection effect of the detection network on a certain category of targets. The larger the value is, the better the overall detection effect will be. The average precision is mainly reflected in the precision–recall curve (also known as the PR curve). In the PR plot, the horizontal axis is the recall rate, which reflects the ability to cover the positive sample, and the value of the vertical axis reflects the precision of predicting the positive sample. The calculation of the average precision is taken as the integral of the precision and recall curve on [0,1]:


(3) $$A_{p}=\int_{0}^{1}P(R)dR$$

The mean of average precision represents the mean of the average precision of all categories in the dataset. It is calculated as the ratio of the sum of the average precision of all categories to the number of all categories:


(4) $$m_{AP}=\frac{\sum A_{P}}{n}$$

The F1 value is a comprehensive evaluation index based on accuracy and recall, which is defined as the average of the harmonization of precision and recall:


(5) $$F_{1}=\frac{2PR}{P+R}\times 100\%$$

### Deep-learning-based detection

To verify the application capacity of Weed25 in weed detection, the YOLO models based on the single-stage detection of convolutional neural networks as well as the two-stage detection of regional convolutional neural network Faster R-CNN were selected as the training algorithm. The main difference was that Faster R-CNN used the most advanced regional recommendation box extraction algorithm Region Proposal Network (RPN). The feature map of the image can be extracted using the feature extraction network. It will be shared by the RPN network and the Faster R-CNN network. Finally, the position of the candidate box was obtained by anchor regression, while the YOLO model transforms object detection into an end-to-end regression problem and improves the detection real-time. The thresholds IoU of 0.5 and batch size of 4 were adjusted for YOLOv3, YOLOv5, and Faster R-CNN. Each model was trained for 100 epochs.

### Test results

**Figure 5:**
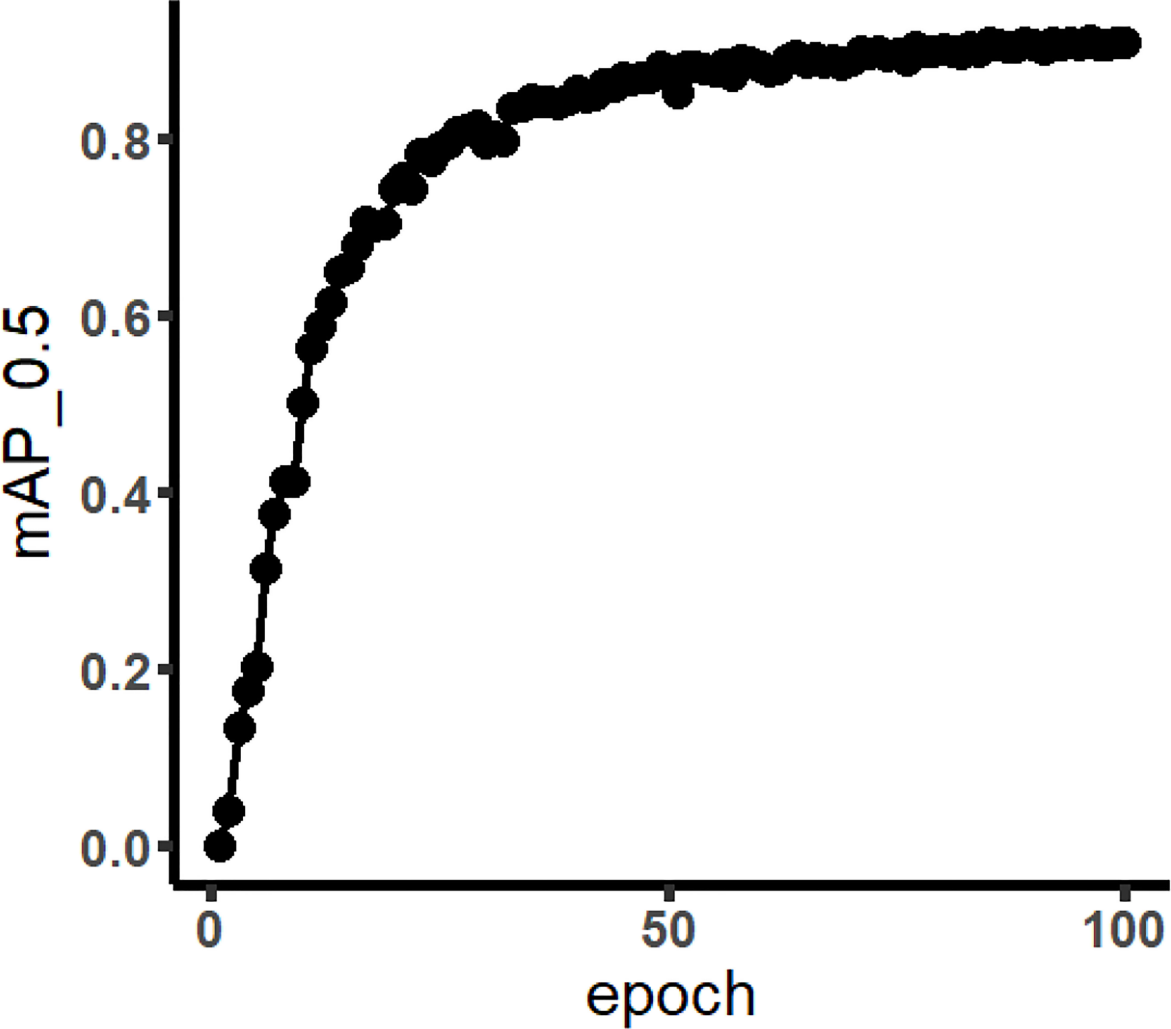
mAP curve of Faster R-CNN (the meaning of mAP_0.5 is that when IoU was set to 0.5, the average precision (AP) of all images in each category would be calculated. Then, the AP of all categories was calculated; that was mAP).

The training results are listed in **Table 4**. It presented that the YOLO model training indicators using Weed 25 were generally acceptable. The difference of mAP between YOLOv5 and YOLOv3 was very small as the values were 92.4% and 91.8%, respectively. The precision was 88.0% and 89.0%, respectively. Moreover, the recall for both reached 99.0%. It showed that Weed25 was available for the YOLO models. For the sake of excluding the advantages of the YOLO model on the training results, Faster R-CNN was employed for the training as well. The results showed that the mAP of Faster R-CNN network was 92.15% (**Figure 5**), which was lower than the mAP of the YOLOv5 networks. It indicated that Weed25 would be capable for precision weed identification model training in future studies.

**Table 4:** Weed identification model training results using YOLO and Faster R-CNN networks with Weed25.

Networks	Precision (%)	Recall (%)	F1 score	Mean average precision (%)
YOLOv3	89.0	99.0	0.88	91.80
YOLOv5	88.0	99.0	0.89	92.40
Faster R-CNN	65.9	98.0	0.78	92.15

**Figure 6:**
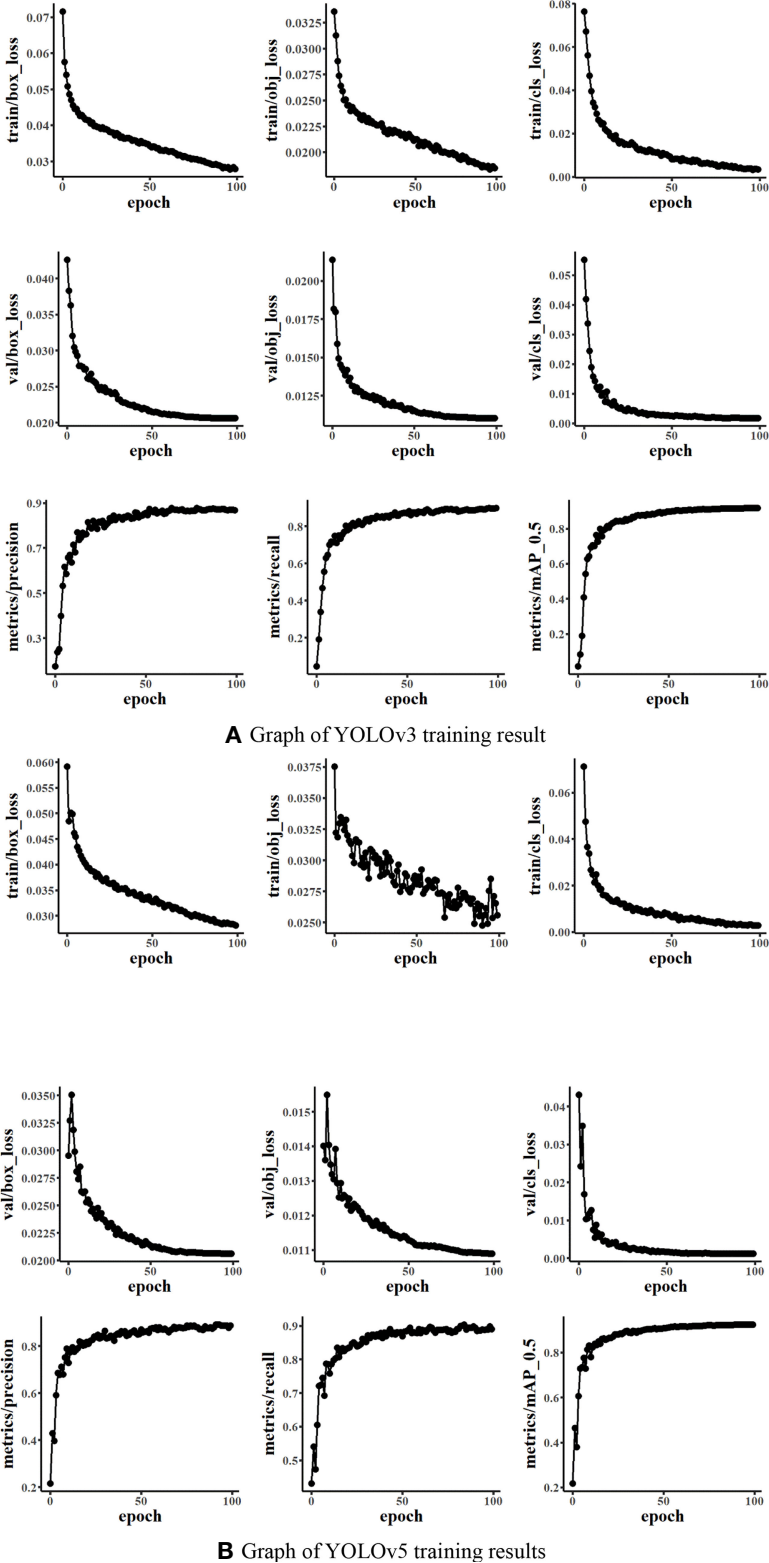
Graph of the You Only Look Once model training results (Train/val box_loss: the bounding box loss of the training dataset or validation dataset; the smaller the box is, the more accurate. Train/val obj_loss: train or val is speculated to be the mean loss of the target detection, and the smaller the target detection is, the more accurate the detection. Train/val cls_loss: train or validation is speculated to be the mean of classification loss, and the smaller the classification is, the more accurate).

**Figure 6** presents the training results of YOLO networks. It could be seen that the box_loss, obj_loss means, and cls_loss means of the training and validation datasets during the training of the model were constantly decreasing. The average precision under mAP_0.5 was constantly increasing. The mAP_0.5 of both YOLOv3 and YOLOv5 was close to 0.9. It indicated that the training effect was good with the dataset Weed25.

**Figure 7:**
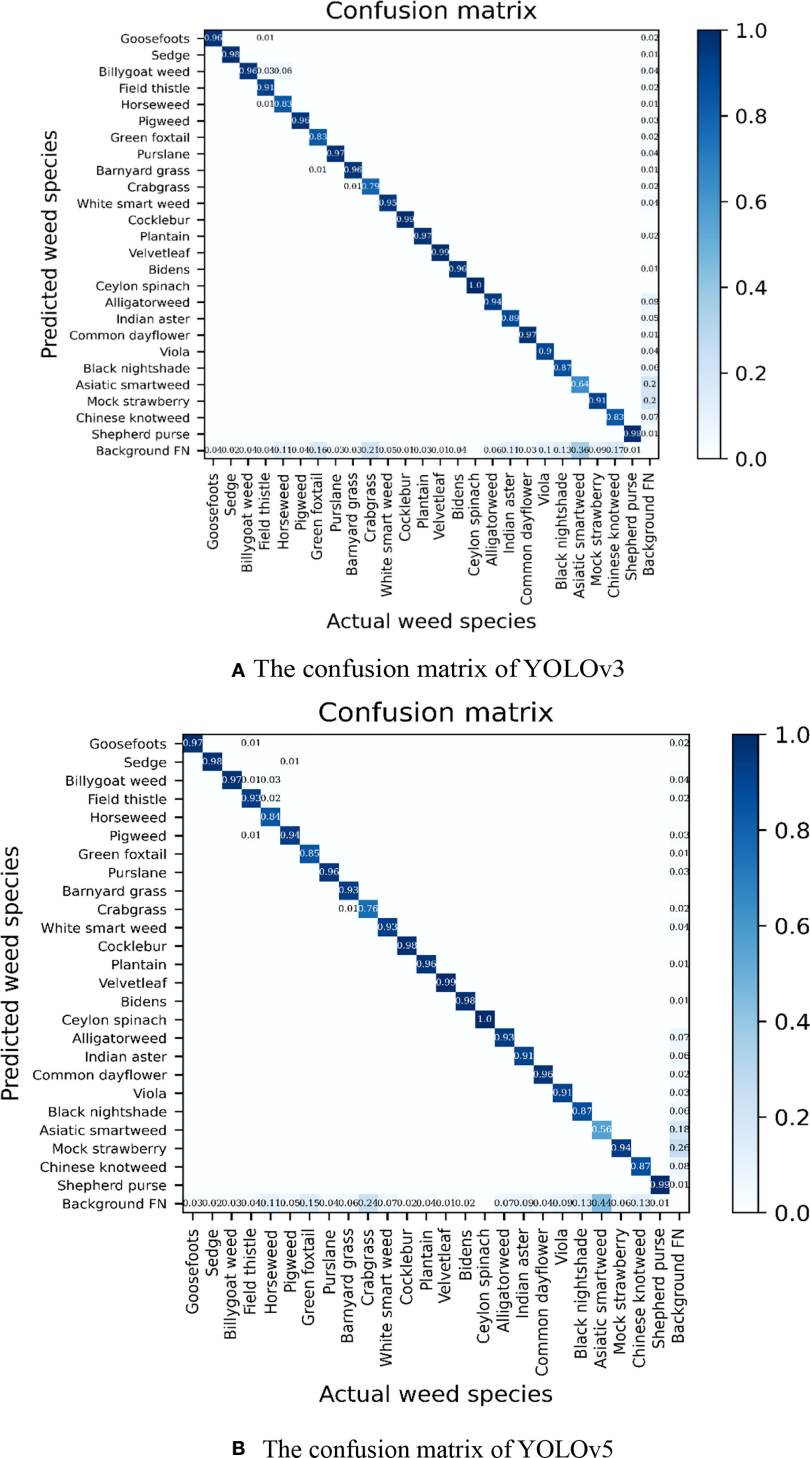
Confusion matrix of You Only Look Once models.

**Figure 8:**
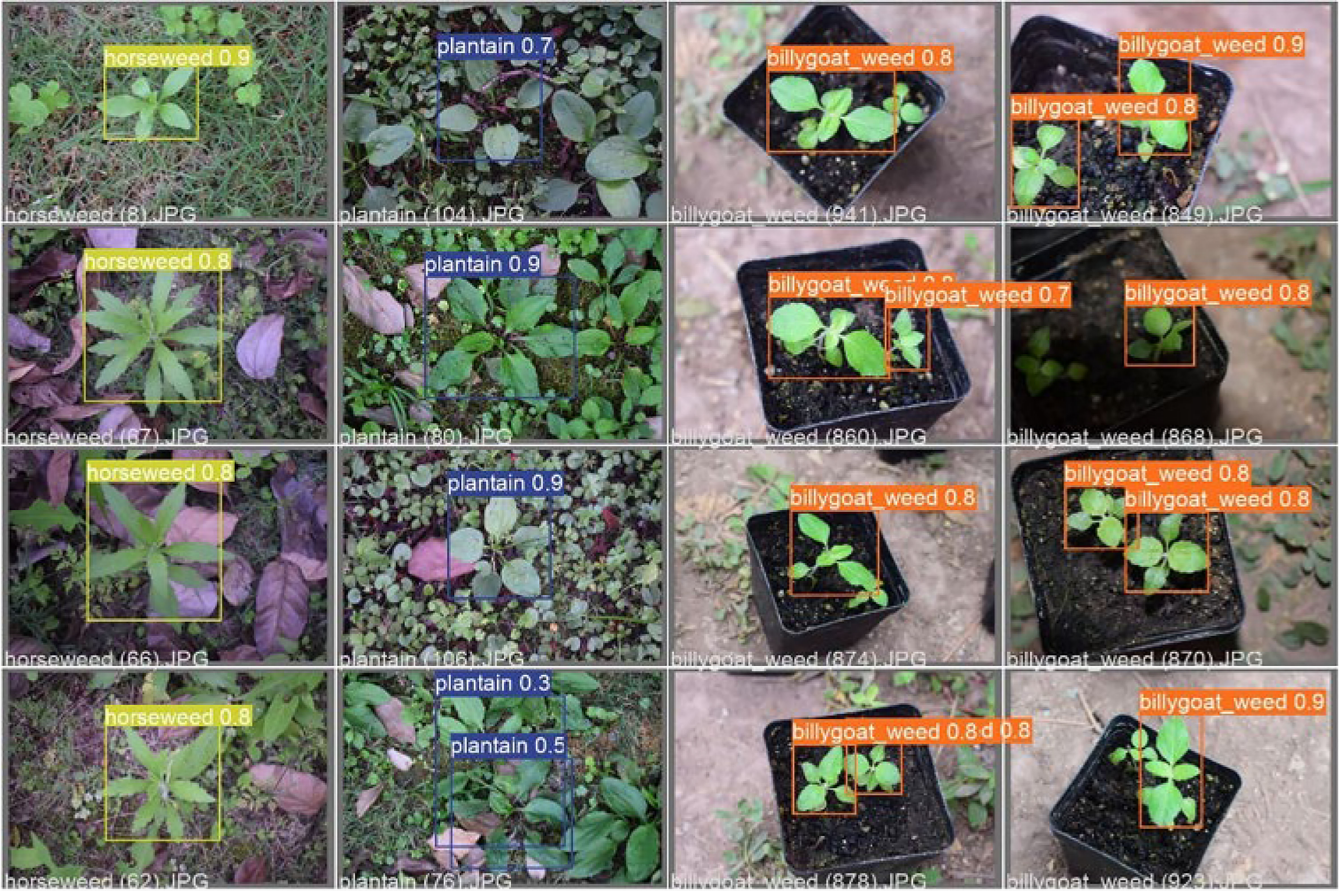
Effect diagram of weed prediction of the You Only Look Once model (in the notes above the detection box, the name of the weed is on the left, and the precision of the weed is on the right).

**Figure 7** presents the confusion matrixes summarizing the identification performance of the YOLOv3 and YOLOv5 models. It could be seen that the classification accuracy of 18 weed species in the YOLOv3 model and 19 weed species in the YOLOv5 model was higher than 0.9. In particular, the identification accuracy of Ceylon spinach reached 1.0. It showed that the model had good recognition capability of this weed. Among the six weed species with a classification accuracy less than 0.9 in the YOLOv5 model, it was found that the classification accuracy of crabgrass and green foxtail in the *Gramineae* family was 0.76 and 0.85, respectively. For the majority of incorrect identification cases, they were predicted as background maps, which showed that the background would have some interference with the detection results. An impressive identification case occurred on the horseweed. With as less as 192 pictures for training and validation in total, the classification accuracy of horseweed reached 0.85. There might be two main reasons that could contribute to this result. Firstly, the appearance features of horseweed were significant. Secondly, the images of the horseweed were not disturbed and occluded by other weeds. Meanwhile, the classification accuracy of Asiatic smartweed with 490 images was as low as 0.56. The features of Asiatic smartweed were not significant as it was a vine weed growing in the soil during the process of collection. On the other hand, the area of soil was larger than the area of weeds in most Asiatic smartweed images. That might be the reason for the incorrect generalization of this weed into the background image. The weed identification results of the YOLO models are shown in **Figure 8**, which displays the identification of weeds in a complex context.

In **Figure 5**, the mAP of training result using Faster R-CNN network is displayed. Faster R-CNN used RPN to generate high-quality regional suggestion boxes, which could effectively improve the identification accuracy of weeds. It was found that the mAP of weeds was 92.15%, with a steady upward trend. The mAP tended to be convergent when the number of iterations was greater than 50. In addition, the identification results of all the weed species are shown in **Table 5**. The AP of Asiatic smartweed was 62.92%, and the precision was only 20%. The average precision of velvetleaf reached 99.70%.

**Table 5:** Training results of Faster R-CNN (precision represents the ratio between the number of correctly detected weeds and predicted weeds of a certain type).

Species	Recall (%)	Precision (%)	F1	Average precision (%)
Alligatorweed	97.14	47.44	0.64	90.31
Asiatic smartweed	90.85	20.00	0.33	62.92
Bidens pilosa	100.00	76.00	0.86	99.26
Black nightshade	97.85	48.15	0.65	95.59
Ceylon spinach	100.00	80.00	0.89	99.69
Chinese knotweed	94.00	29.94	0.45	84.24
Common dayflower	100.00	66.67	0.8	98.95
Indian aster	94.37	33.33	0.49	85.18
Mock strawberry	98.99	30.06	0.46	73.50
Shepherd purse	100.00	71.74	0.84	98.32
Viola	100.00	48.10	0.65	97.13
Velvetleaf	100.00	78.38	0.88	99.70
Barnyard grass	96.36	53.54	0.69	92.80
Billygoat weed	100.00	63.72	0.78	97.73
Cocklebur	100.00	62.73	0.77	98.43
Crabgrass	93.33	39.55	0.56	81.53
Field thistle	100.00	65.17	0.79	98.66
Goosefoots	93.65	60.82	0.74	93.49
Green foxtail	96.30	41.60	0.58	94.62
Horseweed	91.30	63.64	0.75	88.55
Pigweed	96.51	59.29	0.73	93.54
Plantain	97.22	60.87	0.75	97.42
Purslane	98.15	44.54	0.61	97.42
Sedge	96.72	64.84	0.78	95.37
White smartweed	99.02	63.52	0.77	95.48

Recall manifests the proportion of targets for a class of weeds in the sample that were correctly predicted. F1 is defined as the average of the harmonization of precision and recall, and average precision demonstrates the detection effect of the detection network on a certain category of targets.

## Discussion

Although there are many kinds of weeds in the field, we have just collected 25 weed species in this paper. However, many other weeds could also affect crop production. The proposed weed dataset is still insufficient. Therefore, more species of weeds in the crop field will be appended to improve the intelligent weeding technology in the future.

Through the training results of YOLO models and Faster R-CNN, it was found that the AP of weeds such as Asiatic smartweed was not high. For such weeds, more image resources should be collected. Meanwhile, it would be of great significance to take pictures of these weeds avoiding a large-scale background. Because of the narrowness and insignificant appearance features of grasses, the identification of Gramineae weeds was relatively difficult. Quan et al. (2022) used the trained model to identify the broadleaf and Gramineae weeds in the maize fields, resulting in an average accuracy of 94.70% and 87.10%, respectively. It could be seen from the confusion matrix (**Figure 7**) that the predicted values of crabgrass and green foxtail were both also lower than 0.9 in our research. Furthermore, the classification accuracy of crabgrass was just 0.76. Although the classification accuracy of barnyard grass reaches 0.93, barnyard grass could be easily misjudged as crabgrass with the possibility of 0.01. Otherwise, sunlight could also cause some influence on weed identification. Li et al. (2021) collected 1,000 images of weeds on sunny, cloudy, and rainy days. In that study, the parameters of the feature extraction network ResNet-101 were optimized using the feature extraction network. The training results showed that the rate of identification reached 96.02% on sunny days and 90.34% on rainy days. Moreover, the overlapping of leaves was also a main issue, reducing the identification accuracy at present. Pahikkala et al. (2015) identified the species of mixed sativa and dandelions based on different textures. It indicated that leaf textures should be specifically considered when identifying weeds under harsh conditions as overlapping was commonly existing in the images. Thus, factors such as different intensities of illumination and the overlapping leaf have a great impact on weed identification. Methods on overcoming the problems were proposed, but they still lack versatility and robustness (Wang et al., 2019).

While the images of Weed25 were collected under different light intensities, the overlapping leaves and light intensity had not yet been considered for investigation as to their impact on weed classification accuracy. Therefore, subsequent studies would be continued focusing on the investigation of the photography factors which affect the weed classification accuracy. The precise weed identification in farmlands remains a challenge, which limits the development and application of intelligent weeding machines based on deep learning algorithms.

## Conclusion

In this paper, we created a dataset of weeds, Weed25. The images of the dataset were mainly collected from farmlands and lawns. A total of 14,035 images including 25 different weed species were included. Compared with the existing weed dataset, Weed25 contains the weeds that are prevalent in fields. It has the advantages in diversity and average. In addition, YOLOv3, YOLOv5, and Faster R-CNN were employed to train weed identification models using the Weed25 dataset. The average precision was 91.8%, 92.4%, and 92.15% respectively, which indicated that the proposed dataset has the capability for further development of precise weed identification models, which would contribute to the application of intelligent weed control technology in practice.

## Data availability statement

The raw data supporting the conclusions of this article will be made available by the authors without undue reservation.

## Author contributions

PW, YT, LW, and HL contributed to conception and design of the study. YT and FL organized the database. PW, YT, FL, and QN performed deep learning and the statistical analysis. YT wrote the first draft of the manuscript. PW, QN, and HL wrote sections of the manuscript. All authors contributed to the article and approved the submitted version.

## Acknowledgments

This research was funded by the National Natural Science Foundation of China (grant numbers 32201651 and 32001425), the Natural Science Foundation of Chongqing, China (grant numbers cstc2020jcyj-msxmX0459 and cstc2020jcyj-msxmX0414), the Fundamental Research Funds for the Central Universities (SWU-KT22024), and the Open Funding of the Key Laboratory of Modern Agricultural Equipment and Technology (Jiangsu University; grant number MAET202105). The authors would like to appreciate Prof. Dr. Xinping Chen, Prof. Dr. Yunwu Li, Prof. Dr. Wei Qian, Prof. Dr. Nannan Li, Zhantu Zheng, Yu Xia, Long Wan, and Chengrui Xu for technical support.

## Conflict of interest

The authors declare that the research was conducted in the absence of any commercial or financial relationships that could be construed as a potential conflict of interest.

## Publisher’s note

All claims expressed in this article are solely those of the authors and do not necessarily represent those of their affiliated organizations, or those of the publisher, the editors and the reviewers. Any product that may be evaluated in this article, or claim that may be made by its manufacturer, is not guaranteed or endorsed by the publisher.

## References

[B1] Andert S. (2021). The method and timing of weed control affect the productivity of intercropped maize (Zea mays l.) and bean (Phaseolus vulgaris l.). Agriculture 11, 380. doi: 10.3390/agriculture11050380

[B2] Bakhshipour A. Jafari A. (2018). Evaluation of support vector machine and artificial neural networks in weed detection using shape features. Comput. Electron. Agric. 145, 153–160. doi: 10.1016/j.compag.2017.12.032

[B3] Bakhshipour A. Jafari A. Nassiri S. M. Zare D. (2017). Weed segmentation using texture features extracted from wavelet sub-images. Biosyst. Eng. 157, 1–12. doi: 10.1016/j.biosystemseng.2017.02.002

[B4] Bakhshipour A. Zareiforoush H. (2020). Development of a fuzzy model for differentiating peanut plant from broadleaf weeds using image features. Plant Methods 16, 153. doi: 10.1186/s13007-020-00695-1 33292367 PMC7670791

[B5] Cordill C. Grift T. E. (2011). Design and testing of an intra-row mechanical weeding machine for corn. Biosyst. Eng. 110, 247–252. doi: 10.1016/j.biosystemseng.2011.07.007

[B6] Dyrmann M. Karstoft H. Midtiby H. S. (2016). Plant species classification using deep convolutional neural network. Biosyst. Eng. 151, 72–80. doi: 10.1016/j.biosystemseng.2016.08.024

[B7] Everingham M. Van Gool L. Williams C. K. I. Winn J. Zisserman A. (2010). The pascal visual object classes (VOC) challenge. Int. J. Comput. Vis. 88, 303–338. doi: 10.1007/s11263-009-0275-4

[B8] Fan X. Zhou J. Xu Y. Li K. Wen D. (2021). Identification and localization of cotton seedling weeds based on optimized faster r-CNN. J. Agric. Machinery 52, 26–34. doi: 10.6041/jissn.1000-1298.2021.05.003

[B9] Gašparović M. Zrinjski M. Barković Đ. Radočaj D. (2020). An automatic method for weed mapping in oat fields based on UAV imagery. Comput. Electron. Agric. 173, 105385. doi: 10.1016/j.compag.2020.105385

[B10] Giselsson T. M. Jørgensen R. N. Jensen P. K. Dyrmann M. Midtiby H. S. (2017) A public image database for benchmark of plant seedling classification algorithms. Available at: http://arxiv.org/abs/1711.05458 (Accessed May 30, 2022).

[B11] Haug S. Ostermann J. (2015). “A Crop/Weed field image dataset for the evaluation of computer vision based precision agriculture tasks,” in Computer vision - ECCV 2014 workshops lecture notes in computer science. Eds. Agapito L. Bronstein M. M. Rother C. (Cham, Switzerland:Springer International Publishing), 105–116. doi: 10.1007/978-3-319-16220-1_8

[B12] Heap I. (2022) The international herbicide-resistant weed database. Available at: www.weedscience.org.

[B13] Hinton G. E. Osindero S. Teh Y.-W. (2006). A fast learning algorithm for deep belief nets. Neural Comput. 18, 1527–1554. doi: 10.1162/neco.2006.18.7.1527 16764513

[B14] Hun Z. Yuan L. Chen S. (2012). Farmland weed identification primary color atlas (Beijing: China Agriculture Press Co., Ltd).

[B15] Jiang H. Wang P. Zhang Z. Mao W. Zhao B. QI P. (2018). A rapid method for identifying weeds in maize field based on convolutional network and hash code. J. Agric. Machinery 49, 30–38. doi: 10.6041/j.issn.1000-1298.2018.11.004

[B16] Kazmi W. Garcia-Ruiz F. Nielsen J. Rasmussen J. Andersen H. J. (2015). Exploiting affine invariant regions and leaf edge shapes for weed detection. Comput. Electron. Agric. 118, 290–299. doi: 10.1016/j.compag.2015.08.023

[B17] Khan A. Ilyas T. Umraiz M. Mannan Z. I. Kim H. (2020). CED-net: Crops and weeds segmentation for smart farming using a small cascaded encoder-decoder architecture. Electronics 9, 1602. doi: 10.3390/electronics9101602

[B18] Khan S. Tufail M. Khan M. T. Khan Z. A. Anwar S. (2021). Deep learning-based identification system of weeds and crops in strawberry and pea fields for a precision agriculture sprayer. Precis. Agric. 22, 1711–1727. doi: 10.1007/s11119-021-09808-9

[B19] Kunz C. Weber J. F. Peteinatos G. G. Sökefeld M. Gerhards R. (2018). Camera steered mechanical weed control in sugar beet, maize and soybean. Precis. Agric. 19, 708–720. doi: 10.1007/s11119-017-9551-4

[B20] Liebman M. Mohler C. Staver C. (2001). Ecological management of agricultural weeds (New York, USA:Cambridge University Press). doi: 10.1017/CBO9780511541810

[B21] Lin T.-Y. Maire M. Belongie S. Hays J. Perona P. Ramanan D.. (2014). “Microsoft COCO: Common objects in context,” in Computer vision – ECCV 2014 lecture notes in computer science. Eds. Fleet D. Pajdla T. Schiele B. Tuytelaars T. (Cham: Springer International Publishing), 740–755. doi: 10.1007/978-3-319-10602-1_48

[B22] Li K. Xu Y. Zhou J. Fan X. Wei Y. (2021). A seedling-stage weed identification method based on faster r-CNN and data enhancement. J. Xinjiang Univ. (Natural Sci. Edition) (Chinese English) 38, 450–456. doi: 10.13568/j.cnki.651094.651316.2020.06.03.0001

[B23] Marx C. Barcikowski S. Hustedt M. Haferkamp H. Rath T. (2012). Design and application of a weed damage model for laser-based weed control. Biosyst. Eng. 113, 148–157. doi: 10.1016/j.biosystemseng.2012.07.002

[B24] Morin L. (2020). Progress in biological control of weeds with plant pathogens. Annu. Rev. Phytopathol. 58, 201–223. doi: 10.1146/annurev-phyto-010820-012823 32384863

[B25] Olsen A. Konovalov D. A. Philippa B. Ridd P. Wood J. C. Johns J.. (2019). DeepWeeds: A multiclass weed species image dataset for deep learning. Sci Rep 9, 2058. doi: 10.1038/s41598-018-38343-3. arXiv:1810.05726. 30765729 PMC6375952

[B26] Pahikkala T. Kari K. Mattila H. Lepistö A. Teuhola J. Nevalainen O. S.. (2015). Classification of plant species from images of overlapping leaves. Comput. Electron. Agric. 118, 186–192. doi: 10.1016/j.compag.2015.09.003

[B27] Pallottino F. Menesatti P. Figorilli S. Antonucci F. Tomasone R. Colantoni A.. (2018). Machine vision retrofit system for mechanical weed control in precision agriculture applications. Sustainability 10, 2209. doi: 10.3390/su10072209

[B28] Peng M. Xia J. Peng H. (2019). Efficient identification of weeds in cotton field under complex background of faster r-CNN fused with FPN. Trans. Chin. Soc. Agric. Eng. 35, 202–209.

[B29] Peteinatos G. G. Reichel P. Karouta J. Andújar D. Gerhards R. (2020). Weed identification in maize, sunflower, and potatoes with the aid of convolutional neural networks. Remote Sens. 12, 4185. doi: 10.3390/rs12244185

[B30] Quan L. Jiang W. Li H. Li H. Wang Q. Chen L. (2022). Intelligent intra-row robotic weeding system combining deep learning technology with a targeted weeding mode. Biosyst. Eng. 216, 13–31. doi: 10.1016/j.biosystemseng.2022.01.019

[B31] Redmon J. Divvala S. Girshick R. Farhadi A. (2016). “You only look once: Unified, real-time object detection,” in 2016 IEEE conference on computer vision and pattern recognition (CVPR) (Las Vegas, NV, USA: IEEE), 779–788. doi: 10.1109/CVPR.2016.91

[B32] Ren S. He K. Girshick R. Sun J. (2017). Faster r-CNN: Towards real-time object detection with region proposal networks. IEEE Trans. Pattern Anal. Mach. Intell. 39, 1137–1149. doi: 10.1109/TPAMI.2016.2577031 27295650

[B33] Ren P. Li P. Huang L. (2018). Primary color ecological map for identification and control of weeds in farmland (Beijing, China:China Agricultural Science and Technology Press). International Standard Book Number ISBN: 9787511637567.

[B34] Russakovsky O. Deng J. Su H. Krause J. Satheesh S. Ma S.. (2015). ImageNet Large scale visual recognition challenge. Int. J. Comput. Vis. 115, 211–252. doi: 10.1007/s11263-015-0816-y

[B35] Stepanovic S. Datta A. Neilson B. Bruening C. Shapiro C. Gogos G.. (2016). The effectiveness of flame weeding and cultivation on weed control, yield and yield components of organic soybean as influenced by manure application. Renew. Agric. Food Syst. 31, 288–299. doi: 10.1017/S1742170515000216

[B36] Sudars K. Jasko J. Namatevs I. Ozola L. Badaukis N. (2020). Dataset of annotated food crops and weed images for robotic computer vision control. Data Brief 31, 105833. doi: 10.1016/j.dib.2020.105833 32577458 PMC7305380

[B37] Swain K. C. Nørremark M. Jørgensen R. N. Midtiby H. S. Green O. (2011). Weed identification using an automated active shape matching (AASM) technique. Biosyst. Eng. 110, 450–457. doi: 10.1016/j.biosystemseng.2011.09.011

[B38] Tretyakova A. Grudanov N. Kondratkov P. Baranova O. Luneva N. Mysnik Y.. (2020). A database of weed plants in the European part of Russia. Biodiver. Data J. 8, e59176. doi: 10.3897/BDJ.8.e59176 PMC7606405 33192154

[B39] Wang A. Zhang W. Wei X. (2019). A review on weed detection using ground-based machine vision and image processing techniques. Comput. Electron. Agric. 158, 226–240. doi: 10.1016/j.compag.2019.02.005

[B40] Yuan H. Zhao N. Chen M. (2020). Research progress and prospect of field weed recognition based on image processing. J. Agric. Machinery 51, 323–334.

